# Preclinical humanized mouse model with ectopic ovarian tissues

**DOI:** 10.3892/etm.2014.1819

**Published:** 2014-07-01

**Authors:** SHILONG FU, JUE WANG, WU SUN, YI XU, XIAOYU ZHOU, WENJUN CHENG

**Affiliations:** 1 Department of Gynecology, The First Affiliated Hospital of Nanjing Medical University, Nanjing, Jiangsu 210029, P.R. China; 2 Department of General Surgery, The First Affiliated Hospital of Nanjing Medical University, Nanjing, Jiangsu 210029, P.R. China; 3 Department of Clinical Pathology, The First Affiliated Hospital of Nanjing Medical University, Nanjing, Jiangsu 210029, P.R. China; 4 Blood Transfusion, The First Affiliated Hospital of Nanjing Medical University, Nanjing, Jiangsu 210029, P.R. China

**Keywords:** mouse model, preclinical tool, ovarian tissues

## Abstract

The aim of the present study was to establish human ovarian stroma within the mouse subcutaneously, in order for the resulting stroma to serve as a useful preclinical tool to study the progression of human ovarian cancer in a humanized ovarian microenvironment. Normal human ovarian tissues were subcutaneously implanted into severe combined immunodeficient (SCID) mice and then the implants were identified by immunohistochemistry. The implants became vascularized and retained their original morphology for about 4 weeks following implantation. Immunohistochemical staining for cytokeratin-7 confirmed the ovarian origin of the epithelial cells. CD34 staining demonstrated human-derived vessels. Positive estrogen receptor and partially-positive progesterone receptor staining indicated the estrogen and progesterone dependence of the implants. Only vascular pericytes expressed α-smooth muscle actin, indicating the normal ovarian origin of the xenografts. Human ovarian tissue successfully survived in SCID mice and retained its original properties. This humanized mouse model may be used as preclinical tool to investigate ovarian cancer.

## Introduction

Despite rapid advances in the understanding of ovarian carcinogenesis, ovarian cancer remains the most common cause of mortality from gynecological malignancies and the fifth most common cause of cancer mortalities in females in the United States (1,2). Since mouse models of ovarian cancer can mimic the clinical processes of human ovarian cancer, several models have been developed during the past decades to promote *in vivo* research on human ovarian cancer (3–6). However, a major limitation of these models is the lack of a human microenvironment and an inaccurate replication of the interactions between ovarian cancer cells and the human ovarian microenvironment (7,8), which is now known to play a crucial role in the regulation of tumor growth and metastasis (9,10).

To precisely mimic the clinical processes of human cancer, an increasing number of humanized factors have been gradually added to these mouse models. The development of ‘humanized’ mice is now an important research tool for the *in vivo* study of human biological processes (11). Since the first study reported that severe combined immunodeficient (SCID) mice were able to be successfully engrafted with human tissues, normal and neoplastic human tissues have been successfully engrafted into SCID mice (12). The implantation of human tissue xenografts in immunodeficient mice has provided insight into the biology of human cancer, autoimmunity and infectious diseases (11). Proia and Kuperwasser (13) reproducibly established functionally normal breast tissue in mice by implanting reduction mammoplasty tissue samples in an orthotopic xenograft model. The endogenous mouse epithelium was cleared, and comixed human epithelial and stromal cells were implanted to construct a humanized mouse model. However, whether this model may be used to understand normal human breast development or tumorigenesis remains unknown. Bankert *et al* (14) described a humanized mouse model of ovarian cancer that recapitulated the solid tumor progression, ascites formation and metastasis observed in patients. In this model, the tumor and tumor stroma were successfully engrafted into the peritoneal cavity of SCID mice. This model may be used to determine how fibroblasts and lymphocytes within the tumor microenvironment contribute to tumor growth and metastasis. However, this humanized mouse model focused solely upon the engraftment of ovarian cancer tissues, not cancer cells grown in a human microenvironment.

In previous studies, orthotopic implantation of human breast tissues in mice was demonstrated to result in a novel mouse model with a human mammary microenvironment (15), while the implantation of human gastric tissues in mice resulted in a novel mouse model with a human gastric microenvironment (16). To the best of our knowledge, there have been no studies investigating the implantation of normal human ovarian tissue in a SCID mouse model. Therefore, the aim of the present study was to develop a novel protocol for the establishment of human ovarian stroma within a mouse model subcutaneously. It was hypothesized that the resulting stroma may serve as a useful preclinical tool that may allow the progression of human ovarian cancer to be investigated in a humanized ovarian microenvironment.

## Materials and methods

### Animals and materials

A total of 28 female SCID mice (age, 5–6 weeks; C.B-17IcrCrl-scid-bgBR) were purchased from the Model Animal Research Center of Nanjing University (MARC, Nanjing, China). The mice were housed under specific pathogen-free, temperature-controlled conditions. Their cages, bedding and drinking water were autoclaved and changed regularly. Food was sterilized by irradiation. The mice were maintained in a daily cycle of alternating 12-h periods of light and darkness. All experimental procedures were conducted according to the Guide for the Care and Use of Laboratory Animals and were approved by the Animal Care and Use Committee of Nanjing Medical University (Nanjing, China).

Normal, human, noncancerous ovarian tissues were obtained from patients that had undergone a total hysterectomy with prophylactic oophorectomy at the First Affiliated Hospital of Nanjing Medical University. The use of human samples in the study was approved by the Committee for Ethical Review of Research at Nanjing Medical University, according to the ethical guidelines of the Declaration of Helsinki and each patient provided informed consent to participate in the study.

### Implantation of human ovarian tissues in mice

Under sterile conditions, normal human ovarian tissues were sliced into small sections (~4×4×4 mm in size). Three small sections of normal human ovarian tissue were randomly selected for subsequent histological examination; the other pieces were stored in phosphate-buffered saline at 4°C until implantation into the mice.

Prior to implantation, the mice were anesthetized with an intraperitoneal injection of 1% pentobarbital sodium (10 μl/g body weight; Sigma-Aldrich, Steinheim, Germany). The surgical procedure was performed as previously described, with certain modifications (9,10). In brief, 5–6 mm incisions were made using a scalpel on the skin of the left mid-dorsal flank, through which four or five small sections of human ovarian tissue were subcutaneously implanted. The procedure was finished within 6 h following the prophylactic oophorectomy. All the mice received gentamicin in the drinking water (800,000 U/l) for up to one week following implantation. A total of 14 SCID mice were implanted with xenografts from one individual patient, while an additional 14 SCID mice were implanted with xenografts from two patients in this phase of the study where the xenografts come from three human patients.

### Gross observation and specimen collection

Following implantation, the ovarian xenografts were subjected to weekly gross examinations. Mice were sacrificed at one, two or four weeks following surgery, and the human xenografts, including the mouse tissue surrounding the implanted human ovarian tissues, were harvested for histological assessment and immunohistochemical analysis. The harvested specimens were fixed in 10% formalin for examination.

### Histological and immunohistochemical examination

Specimens were dehydrated and embedded in paraffin. Sections (4-μm thick) were stained with hematoxylin and eosin and examined under a microscope. For immunohistochemical staining, the specimens were deparaffinized and rehydrated using xylene and graded alcohol. Sections were mounted on slides, subjected to antigen retrieval and then incubated with rabbit monoclonal antibodies against human estrogen receptor (ER), progesterone receptor (PR) and CD34 (Maxim Biotech, San Francisco, CA, USA), as well as human cytokeratin (CK)-7, CK-20 and α-smooth muscle actin (CK-7, CK-20, α-SMA; LabVision, Kalamazoo, MI, USA). Immunocomplexes were visualized using the diaminobenzidine method and sections were counterstained with hematoxylin. Negative controls were serial sections processed without the primary antibody. These reagents exhibited no cross-reactivity between the relevant species (mouse and human). All histological examinations were performed by one experienced pathologist.

## Results

### Acceptance of xenografts in SCID mice

**Figure 1:**
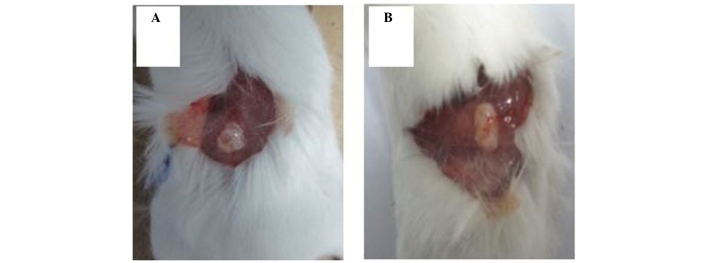
Gross observations of the subcutaneous xenografts of human ovarian tissues in severe combined immunodeficient mice revealed that (A) subcutaneous xenografts survived well, and (B) marked vascularization of the xenografts was present.

From one week following the implantation, the ovarian xenografts were subjected to weekly gross examinations. The embedded human ovarian tissues survived in the SCID mouse hosts for up to six weeks. No infection or tissue rejection reactions were observed at the time of tissue harvest, and all the implants became vascularized and survived well on the sheath of mouse muscles in the subcutaneous tissue (Fig. 1A and B).

### Histological examination

**Figure 2:**
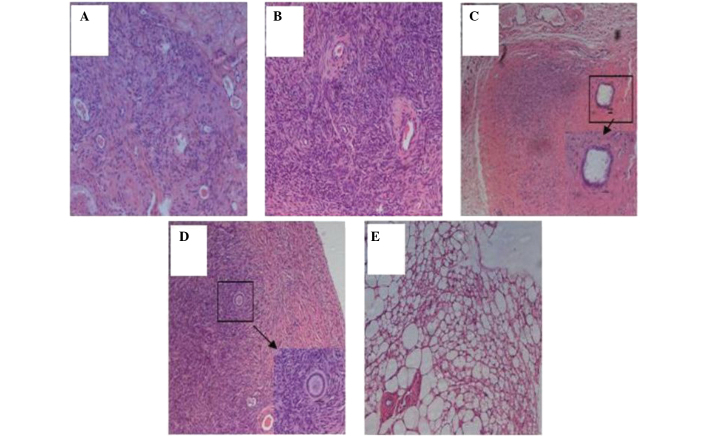
Representative photomicrographs of human ovarian tissues stained with hematoxylin and eosin. (A) Xenografts show a normal structure two weeks following subcutaneous transplantation into a severe combined immunodeficient (SCID) mouse (magnification, ×200). (B) Normal ovarian tissue structure prior to transplantation (magnification, ×200). (C) Follicular architecture in the implanted ovarian tissues two weeks following subcutaneous transplantation into a SCID mouse (magnification, ×100; inset magnification, ×400). (D) Follicular architecture in ovarian tissue prior to transplantation (magnification, ×100; inset magnification, ×400). (E) No definite morphological pattern was observed in the subcutaneous xenografts of human ovarian tissue in the nude mice (magnification, ×200).

Histological evaluation revealed that the morphology of the human ovarian tissues embedded in the SCID mice was similar to the morphology of the original tissues prior to implantation. The viability of the cells within the xenografts was confirmed by the intact nature of the cell membranes, normal granulation of the cytoplasm and normal size and staining characteristics of the nucleus (Fig. 2A and B). In a number of the engrafted ovarian tissues, which were derived from a perimenopausal female, the follicular architecture of the original tissues was retained (Fig. 2C and D). However, ovarian tissues embedded in the nude mice in the preliminary experiments were unable to survive, and histological evaluation indicated that the specific morphology of the ovarian tissues was not retained in the implanted tissues (Fig. 2E).

### Immunohistochemical analysis

**Figure 3:**
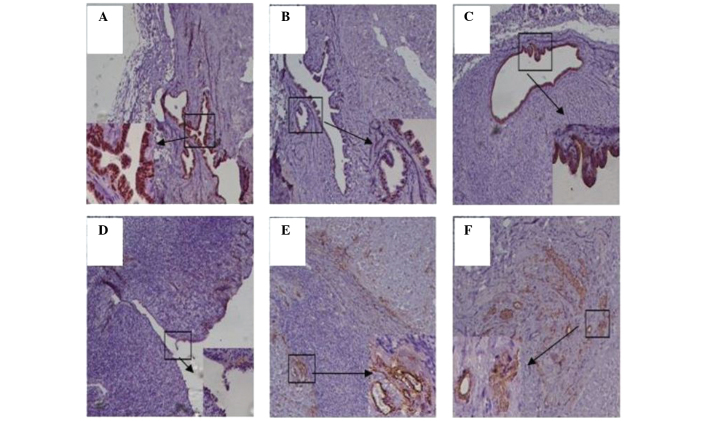
Immunohistochemical analysis of human ovarian tissues implanted in severe combined immunodeficient mice. (A) Positive staining for the estrogen receptor in implanted human ovarian tissue (magnification, ×100; inset magnification, ×400). (B) Partially positive staining for the progesterone receptor in implanted human ovarian tissue (magnification, ×100; inset magnification, ×400). (C) Positive staining for cytokeratin (CK)-7 in implanted human ovarian tissue, indicating that the normal ovarian architecture was retained (magnification, ×100; inset magnification, ×400). (D) Positive staining for CK-7 in normal ovarian tissue prior to transplantation (magnification, ×100; inset magnification, ×400). (E) Positive staining for CD34 in the implanted human ovarian tissue (magnification, ×100; inset magnification, ×400). (F) Positive staining for α-smooth muscle actin in the implanted human ovarian tissue (magnification, ×100; inset magnification, ×400).

Immunohistochemical analysis demonstrated that the harvested specimens were positive for ER and partially positive for PR, indicating that the inoculated ovarian tissues had remained estrogen- and progesterone-dependent, similar to the original tissues (Fig. 3A and B). Positive staining was also observed for anti-human CK-7, indicating that the majority of the ovarian epithelium in the original and transplanted tissues was derived from the human ovarian tissues (Fig. 3C and D). Positive staining for anti-human CD34 indicated that the implanted ovarian tissues were well vascularized and remained viable (Fig. 3E). In addition, α-SMA, which is often expressed in cancer-associated fibroblasts, was only detected in the vascular pericytes and not in the ovarian epithelium or ovarian stroma, indicating that the transplanted tissues were normal ovarian tissues similar to the original tissue (Fig. 3F).

## Discussion

Complex biological processes often require *in vivo* analysis, and a number of important research advances have been made using mice as a model for the study of various biological systems. However, mice are not the same as humans, and *in vivo* studies of human biology are severely limited by ethical and technical constraints. There is a growing need for animal models, which can be used to construct *in vivo* studies of human cells, tissues and organs without putting individuals at risk. Humanized mice, or mouse-human chimeras, have been developed to overcome these constraints and are now an important research tool for the *in vivo* study of human cells and tissues (11). Humanized mice are defined as immunodeficient mice engrafted with hematopoietic cells or tissues, or mice that transgenically express human genes (11). The development of mice that are ‘humanized’ by the engraftment of human tissues provides an opportunity for the *in vivo* study of human biological processes, which may otherwise not be possible. In the present study, a novel protocol was developed for the establishment of normal human ovarian stroma within the subcutaneous tissues of mice. It was hypothesized that the implanted stroma would accurately mimic the human ovarian microenvironment, and result in a humanized mouse model that may potentially serve as a useful preclinical tool for investigating the progression of human ovarian cancer.

A key factor in the success of this engraftment and the long-term survival of human xenografts is the use of SCID mice. Failure to develop mature T and B lymphocytes and the lack of an immune system makes SCID mice an ideal model for cell and tissue-transfer experiments. As a classical model, the subcutaneous mouse model has been widely used for a number of years. In the present study, normal, human ovarian tissues were subcutaneously implanted into SCID mice with the aim of developing a novel human ovarian tissue-derived mouse model.

Numerous studies have used mice with xenografts implanted under the renal capsule (17). However, in the present study, human ovarian tissue was embedded subcutaneously in the SCID mice. The advantages of subcutaneous implantation include a plentiful blood supply at the implantation site and minimal surgical damage to the tissues. Between day 7 and 14 following implantation, neovascularization was observed in the implanted tissue. This model has the potential to improve the understanding of the crosstalk between tissue stroma and the epithelium, as well as the factors involved in tumor initiation and progression. The humanized ovarian microenvironment established in this mouse model offers a basis to study the proliferation of human ovarian cancer cells in a human ovarian milieu.

A normal, human tissue microenvironment contains three-dimensional intercellular interactions between the stroma cells, epithelial cells and cytokines. These are essential for maintaining epithelial polarity and modulating the growth inhibition of normal cells (18,19). However, when cancer cells emerge in normal tissue, they produce a range of stroma-modulating factors to activate the stromal cells in their microenvironment. The activated stromal cells then secrete matrix metalloproteinases and cytokines to promote tumor growth, stimulate angiogenesis, increase inflammatory responses and induce cell differentiation (20–22). Therefore, the normal tissue microenvironment is disrupted and transforms into the tumorous tissue microenvironment.

In the present study, the epithelial cells in the xenografts were shown to be derived from normal ovarian tissues, as these cells were positive for CK-7, which is diffusely distributed throughout the cytoplasm of ovarian cells. Furthermore, human-derived vessels within the implants remained viable for a period of four weeks following implantation. Positive staining for the ER and partially positive staining for the PR indicated that the inoculated ovarian tissues continued to be estrogen- and progesterone-dependent. α-SMA was found to be expressed only in the vascular pericytes and not in the epithelium or stromal fibroblasts, indicating that the subcutaneous xenografts of human ovarian tissues in the SCID mice consisted of cells derived from normal tissues. These observations indicated that the implanted human ovarian tissues survived well in the SCID mice.

The results of the present study indicate that the humanized SCID mouse ovarian transplant model has the potential to serve as a useful preclinical tool, which may be used to build a humanized mouse model of ovarian cancer to investigate the efficacy of chemotherapeutic strategies. The absence of an immune system is one of the major limitations inherent in this type of model. However, SCID mice are likely to continue to be critical in establishing xenograft models that recapitulate the tumor growth and spread observed in patients.

In conclusion, the present study demonstrated that human ovarian tissue successfully survives in a SCID mouse host and retains the properties of the original normal ovarian tissues. In addition, the experimental protocol used in the current study may be used to establish a humanized mouse model of ovarian cancer for preclinical research.

## Abbreviations

SCID: severe combined immunodeficient
ER: estrogen receptor
PR: progesterone receptor
CK: cytokeratin
α-SMA: α-smooth muscle actin
